# (2,2′-Biquinoline-κ^2^ *N*,*N*′)dibromidopalladium(II)

**DOI:** 10.1107/S1600536812015425

**Published:** 2012-04-18

**Authors:** Kwang Ha

**Affiliations:** a School of Applied Chemical Engineering, The Research Institute of Catalysis, Chonnam National University, Gwangju 500-757, Republic of Korea

## Abstract

The Pd^II^ ion in the title complex, [PdBr_2_(C_18_H_12_N_2_)], is four-coordinated in a distorted square-planar environment by the two N atoms from the chelating 2,2′-biquinoline (Biqu) ligand and two mutually *cis* Br^−^ anions. The Biqu ligand is not planar, the dihedral angle between the quinoline systems being 17.2 (2)°. In the crystal, the complex mol­ecules are connected by C—H⋯Br hydrogen bonds, forming chains along the *c* axis. When viewed down the *b* axis, successive chains are stacked in opposite directions. Intra­molecular C—H⋯Br hydrogen bonds are also observed.

## Related literature

For the crystal structure of the related chlorido Pd^II^ complex [PdCl_2_(Biqu)], see: Muranishi *et al.* (2005 ▶).
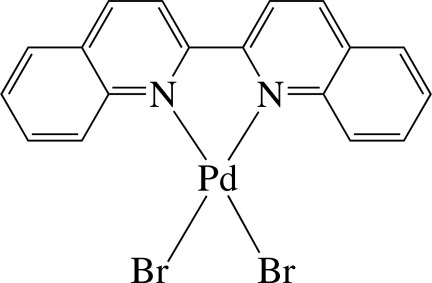


## Experimental

### 

#### Crystal data

[PdBr_2_(C_18_H_12_N_2_)]
*M* *_r_* = 522.50
Triclinic, 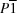

*a* = 8.9390 (5) Å
*b* = 9.2187 (5) Å
*c* = 11.1486 (6) Å
α = 72.398 (1)°
β = 69.318 (1)°
γ = 87.258 (1)°
*V* = 817.47 (8) Å^3^
*Z* = 2
Mo *K*α radiation
μ = 6.02 mm^−1^
*T* = 200 K
0.17 × 0.12 × 0.11 mm

#### Data collection

Bruker SMART 1000 CCD diffractometer
Absorption correction: multi-scan (*SADABS*; Bruker, 2000 ▶) *T*_min_ = 0.813, *T*_max_ = 1.000
5100 measured reflections
3126 independent reflections
2612 reflections with *I* > 2σ(*I*)
*R*_int_ = 0.018

#### Refinement

*R*[*F* ^2^ > 2σ(*F* ^2^)] = 0.028
*wR*(*F* ^2^) = 0.070
*S* = 1.12
3126 reflections
208 parameters
H-atom parameters constrained
Δρ_max_ = 0.67 e Å^−3^
Δρ_min_ = −0.65 e Å^−3^

### 

Data collection: *SMART* (Bruker, 2000 ▶); cell refinement: *SAINT* (Bruker, 2000 ▶); data reduction: *SAINT*; program(s) used to solve structure: *SHELXS97* (Sheldrick, 2008 ▶); program(s) used to refine structure: *SHELXL97* (Sheldrick, 2008 ▶); molecular graphics: *ORTEP-3* (Farrugia, 1997 ▶) and *PLATON* (Spek, 2009 ▶); software used to prepare material for publication: *SHELXL97*.

## Supplementary Material

Crystal structure: contains datablock(s) global, I. DOI: 10.1107/S1600536812015425/rk2351sup1.cif

Structure factors: contains datablock(s) I. DOI: 10.1107/S1600536812015425/rk2351Isup2.hkl

Additional supplementary materials:  crystallographic information; 3D view; checkCIF report

## Figures and Tables

**Table 1:** Hydrogen-bond geometry (Å, °)

*D*—H⋯*A*	*D*—H	H⋯*A*	*D*⋯*A*	*D*—H⋯*A*
C2—H2⋯Br1	0.95	2.73	3.252 (5)	116
C14—H14⋯Br1^i^	0.95	2.90	3.754 (5)	150
C17—H17⋯Br2	0.95	2.85	3.261 (5)	107

Symmetry code: (i) 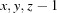.

**Fig. 1.**
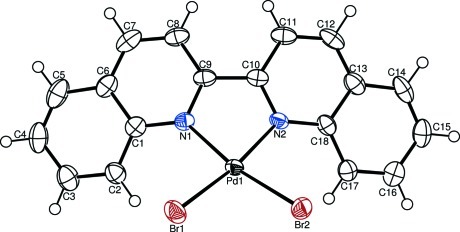
A molecular structure of the title complex with the atom numbering scheme. Displacement ellipsoids are drawn at the 50% probability level. H atoms are presented as a small spheres of arbitrary radius.

**Fig. 2.**
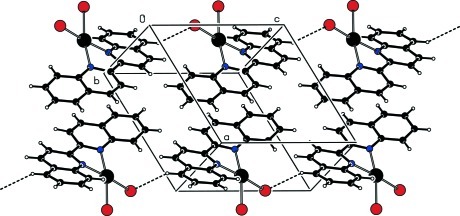
A view of the unit-cell contents of the title complex. Intermolecular C–H···Br H-bond interactions are drawn with dashed lines.

## supplementary crystallographic information

### Comment

The title complex, [PdBr_2_(C_18_H_12_N_2_)], crystallized in the triclinic space group *P*1, whereas the analogous chlorido Pd^II^ complex [PdCl_2_(C_18_H_12_N_2_)] crystallized in the monoclinic space group *P*2_1_/*c* (Muranishi *et al.*, 2005).

The central Pd^II^ ion is four-coordinated in a distorted square-planar environment by the two N atoms from the chelating 2,2'-biquinoline (Biqu) ligand and two mutually *cis* Br^-^ anions (Fig. 1). The main contribution to the distortion is the tight N1-Pd1-N2 chelate angle of 78.90 (15)°, which results in non-linear *trans* axes [angle Br1–Pd1-N2 = 169.85 (10)° and angle Br2–Pd1-N1 = 167.99 (11)°]. The pairs of Pd-N and Pd–Br bond lengths are nearly equivalent [Pd-N = 2.064 (4)Å and 2.073 (4)Å; Pd–Br = 2.4113 (6)Å and 2.4151 (6)Å]. In the crystal structure, the Biqu ligand is not planar. The dihedral angle between the least-squares planes of the quinoline rings is 17.2 (2)°. The quinoline rings are inclined considerably to the least-squares plane of the PdBr_2_N_2_ unit [maximum deviation = 0.162 (1)Å], making dihedral angles of 41.46 (8)° and 44.33 (8)°. In the crystal, the complex molecules are connected by intermolecular C–H···Br hydrogen bonds, forming chains along the *c* axis (Fig. 2 and Table 1). When viewed down the *b* axis, successive chains are stacked in opposite directions. Intramolecular C–H···Br hydrogen bonds are also observed (Table 1). In addition, intermolecular π···π interactions between the six-membered rings are present, the shortest ring centroid-centroid distance being 3.753 (3)Å between pyridine rings.

### Experimental

To a solution of K_2_PdBr_4_ (0.1507 g, 0.299 mmol) in *Me*OH (20 ml) was added 2,2'-biquinoline (0.0772 g, 0.301 mmol) and stirred for 3 h at room temperature. After addition of H_2_O (30 ml) to the reaction mixture, the formed precipitate was separated by filtration and washed with H_2_O and acetone, and dried at 323 K, to give a pale red powder (0.1305 g). Crystals suitable for X-ray analysis were obtained by slow evaporation from an acetone solution.

### Refinement

H atoms were positioned geometrically and allowed to ride on their respective parent atoms: C–H = 0.95Å with *U*_iso_(H) = 1.2*U*_eq_(C). The highest peak (0.67eÅ^-3^) and the deepest hole (-0.65eÅ^-3^) in the difference Fourier map are located 0.86Å and 0.84Å, respectively, from the atoms H14 and Pd1.

### Crystal data

**Table:**

[PdBr_2_(C_18_H_12_N_2_)]	*Z* = 2
*M**_r_* = 522.50	*F*(000) = 500
Triclinic, *P*1	*D*_x_ = 2.123 Mg m^−^^3^
Hall symbol: -P 1	Mo *K*α radiation, λ = 0.71073 Å
*a* = 8.9390 (5) Å	Cell parameters from 3201 reflections
*b* = 9.2187 (5) Å	θ = 2.6–26.0°
*c* = 11.1486 (6) Å	µ = 6.02 mm^−^^1^
α = 72.398 (1)°	*T* = 200 K
β = 69.318 (1)°	Block, red
γ = 87.258 (1)°	0.17 × 0.12 × 0.11 mm
*V* = 817.47 (8) Å^3^	

### Data collection

**Table:**

Bruker SMART 1000 CCD diffractometer	3126 independent reflections
Radiation source: fine-focus sealed tube	2612 reflections with *I* > 2σ(*I*)
Graphite monochromator	*R*_int_ = 0.018
φ and ω scans	θ_max_ = 26.0°, θ_min_ = 2.1°
Absorption correction: multi-scan (*SADABS*; Bruker, 2000)	*h* = −10→11
*T*_min_ = 0.813, *T*_max_ = 1.000	*k* = −11→10
5100 measured reflections	*l* = −13→13

### Refinement

**Table:**

Refinement on *F*^2^	Primary atom site location: structure-invariant direct methods
Least-squares matrix: full	Secondary atom site location: difference Fourier map
*R*[*F*^2^ > 2σ(*F*^2^)] = 0.028	Hydrogen site location: inferred from neighbouring sites
*wR*(*F*^2^) = 0.070	H-atom parameters constrained
*S* = 1.12	*w* = 1/[σ^2^(*F*_o_^2^) + (0.0152*P*)^2^ + 2.2618*P*] where *P* = (*F*_o_^2^ + 2*F*_c_^2^)/3
3126 reflections	(Δ/σ)_max_ < 0.001
208 parameters	Δρ_max_ = 0.67 e Å^−^^3^
0 restraints	Δρ_min_ = −0.65 e Å^−^^3^

### Special details

**Table:**

Geometry. All s.u.'s (except the s.u. in the dihedral angle between two l.s. planes) are estimated using the full covariance matrix. The cell s.u.'s are taken into account individually in the estimation of s.u.'s in distances, angles and torsion angles; correlations between s.u.'s in cell parameters are only used when they are defined by crystal symmetry. An approximate (isotropic) treatment of cell s.u.'s is used for estimating s.u.'s involving l.s. planes.
Refinement. Refinement of *F*^2^ against ALL reflections. The weighted *R*-factor *wR* and goodness of fit *S* are based on *F*^2^, conventional *R*-factors *R* are based on *F*, with *F* set to zero for negative *F*^2^. The threshold expression of *F*^2^ > σ(*F*^2^) is used only for calculating *R*-factors(gt) *etc*. and is not relevant to the choice of reflections for refinement. *R*-factors based on *F*^2^ are statistically about twice as large as those based on *F*, and *R*-factors based on ALL data will be even larger.

### Fractional atomic coordinates and isotropic or equivalent isotropic displacement parameters (Å^2^)

**Table:**

	*x*	*y*	*z*	*U*_iso_*/*U*_eq_	
Pd1	0.18588 (4)	0.23543 (4)	0.12682 (3)	0.02511 (10)	
Br1	0.27983 (6)	0.30923 (6)	0.27917 (5)	0.03917 (15)	
Br2	0.46432 (6)	0.24992 (6)	−0.01469 (5)	0.03636 (14)	
N1	−0.0535 (4)	0.2651 (4)	0.2177 (4)	0.0272 (8)	
N2	0.0935 (4)	0.2114 (4)	−0.0130 (4)	0.0262 (8)	
C1	−0.1396 (6)	0.2604 (5)	0.3488 (5)	0.0298 (11)	
C2	−0.0886 (6)	0.1742 (6)	0.4531 (5)	0.0355 (11)	
H2	0.0083	0.1239	0.4333	0.043*	
C3	−0.1800 (7)	0.1634 (6)	0.5841 (5)	0.0429 (13)	
H3	−0.1471	0.1023	0.6546	0.051*	
C4	−0.3222 (7)	0.2412 (7)	0.6164 (6)	0.0492 (15)	
H4	−0.3821	0.2355	0.7073	0.059*	
C5	−0.3710 (7)	0.3236 (7)	0.5160 (6)	0.0488 (15)	
H5	−0.4661	0.3762	0.5375	0.059*	
C6	−0.2853 (6)	0.3342 (6)	0.3796 (5)	0.0340 (11)	
C7	−0.3393 (6)	0.4086 (6)	0.2740 (6)	0.0428 (13)	
H7	−0.4315	0.4659	0.2909	0.051*	
C8	−0.2613 (6)	0.3996 (5)	0.1484 (6)	0.0350 (12)	
H8	−0.3032	0.4427	0.0789	0.042*	
C9	−0.1173 (6)	0.3254 (5)	0.1219 (5)	0.0297 (11)	
C10	−0.0315 (5)	0.2984 (5)	−0.0078 (5)	0.0267 (10)	
C11	−0.0841 (6)	0.3520 (5)	−0.1181 (5)	0.0358 (12)	
H11	−0.1739	0.4125	−0.1125	0.043*	
C12	−0.0034 (6)	0.3152 (6)	−0.2328 (5)	0.0386 (13)	
H12	−0.0303	0.3588	−0.3108	0.046*	
C13	0.1192 (6)	0.2134 (6)	−0.2371 (5)	0.0367 (12)	
C14	0.1985 (7)	0.1614 (7)	−0.3494 (5)	0.0426 (14)	
H14	0.1746	0.2013	−0.4292	0.051*	
C15	0.3082 (7)	0.0553 (6)	−0.3442 (6)	0.0432 (13)	
H15	0.3597	0.0206	−0.4200	0.052*	
C16	0.3463 (6)	−0.0038 (6)	−0.2269 (5)	0.0390 (12)	
H16	0.4214	−0.0800	−0.2235	0.047*	
C17	0.2771 (6)	0.0469 (5)	−0.1187 (5)	0.0327 (11)	
H17	0.3049	0.0066	−0.0408	0.039*	
C18	0.1640 (6)	0.1591 (5)	−0.1213 (5)	0.0293 (10)	

### Atomic displacement parameters (Å^2^)

**Table:**

	*U*^11^	*U*^22^	*U*^33^	*U*^12^	*U*^13^	*U*^23^
Pd1	0.02232 (19)	0.03082 (19)	0.02356 (19)	0.00450 (14)	−0.01142 (15)	−0.00677 (15)
Br1	0.0325 (3)	0.0603 (3)	0.0312 (3)	0.0010 (2)	−0.0168 (2)	−0.0163 (3)
Br2	0.0241 (3)	0.0522 (3)	0.0343 (3)	0.0056 (2)	−0.0098 (2)	−0.0166 (2)
N1	0.021 (2)	0.030 (2)	0.031 (2)	0.0009 (16)	−0.0103 (17)	−0.0085 (17)
N2	0.023 (2)	0.031 (2)	0.023 (2)	0.0006 (16)	−0.0107 (16)	−0.0026 (17)
C1	0.024 (2)	0.031 (2)	0.037 (3)	−0.003 (2)	−0.011 (2)	−0.012 (2)
C2	0.034 (3)	0.040 (3)	0.031 (3)	−0.001 (2)	−0.011 (2)	−0.008 (2)
C3	0.041 (3)	0.049 (3)	0.032 (3)	−0.009 (3)	−0.007 (2)	−0.008 (3)
C4	0.042 (3)	0.061 (4)	0.035 (3)	−0.009 (3)	0.006 (3)	−0.021 (3)
C5	0.028 (3)	0.053 (3)	0.062 (4)	−0.002 (3)	0.000 (3)	−0.032 (3)
C6	0.021 (2)	0.035 (3)	0.043 (3)	−0.002 (2)	−0.006 (2)	−0.015 (2)
C7	0.029 (3)	0.038 (3)	0.067 (4)	0.010 (2)	−0.017 (3)	−0.026 (3)
C8	0.028 (3)	0.032 (3)	0.050 (3)	0.004 (2)	−0.021 (2)	−0.012 (2)
C9	0.028 (3)	0.026 (2)	0.041 (3)	0.002 (2)	−0.020 (2)	−0.010 (2)
C10	0.026 (2)	0.023 (2)	0.033 (3)	−0.0025 (19)	−0.015 (2)	−0.005 (2)
C11	0.033 (3)	0.034 (3)	0.048 (3)	0.001 (2)	−0.027 (3)	−0.008 (2)
C12	0.046 (3)	0.042 (3)	0.032 (3)	−0.008 (3)	−0.026 (3)	−0.001 (2)
C13	0.037 (3)	0.043 (3)	0.030 (3)	−0.006 (2)	−0.015 (2)	−0.007 (2)
C14	0.046 (3)	0.058 (4)	0.023 (3)	−0.018 (3)	−0.013 (2)	−0.007 (2)
C15	0.040 (3)	0.053 (3)	0.037 (3)	−0.007 (3)	−0.004 (3)	−0.025 (3)
C16	0.035 (3)	0.038 (3)	0.045 (3)	−0.005 (2)	−0.009 (2)	−0.018 (3)
C17	0.033 (3)	0.035 (3)	0.030 (3)	0.000 (2)	−0.010 (2)	−0.011 (2)
C18	0.031 (3)	0.033 (3)	0.027 (3)	−0.005 (2)	−0.013 (2)	−0.009 (2)

### Geometric parameters (Å, º)

**Table:**

Pd1—N1	2.064 (4)	C7—H7	0.9500
Pd1—N2	2.073 (4)	C8—C9	1.407 (7)
Pd1—Br1	2.4113 (6)	C8—H8	0.9500
Pd1—Br2	2.4151 (6)	C9—C10	1.468 (7)
N1—C9	1.349 (6)	C10—C11	1.413 (6)
N1—C1	1.378 (6)	C11—C12	1.363 (7)
N2—C10	1.339 (6)	C11—H11	0.9500
N2—C18	1.369 (6)	C12—C13	1.405 (7)
C1—C2	1.405 (7)	C12—H12	0.9500
C1—C6	1.421 (7)	C13—C14	1.415 (7)
C2—C3	1.373 (7)	C13—C18	1.425 (6)
C2—H2	0.9500	C14—C15	1.355 (8)
C3—C4	1.416 (8)	C14—H14	0.9500
C3—H3	0.9500	C15—C16	1.411 (8)
C4—C5	1.350 (8)	C15—H15	0.9500
C4—H4	0.9500	C16—C17	1.357 (7)
C5—C6	1.415 (7)	C16—H16	0.9500
C5—H5	0.9500	C17—C18	1.413 (7)
C6—C7	1.404 (7)	C17—H17	0.9500
C7—C8	1.352 (7)		
			
N1—Pd1—N2	78.90 (15)	C7—C8—C9	119.2 (5)
N1—Pd1—Br1	96.71 (11)	C7—C8—H8	120.4
N2—Pd1—Br1	169.85 (10)	C9—C8—H8	120.4
N1—Pd1—Br2	167.99 (11)	N1—C9—C8	121.8 (5)
N2—Pd1—Br2	96.02 (11)	N1—C9—C10	114.9 (4)
Br1—Pd1—Br2	86.49 (2)	C8—C9—C10	123.1 (4)
C9—N1—C1	119.3 (4)	N2—C10—C11	121.6 (4)
C9—N1—Pd1	109.1 (3)	N2—C10—C9	116.2 (4)
C1—N1—Pd1	130.2 (3)	C11—C10—C9	122.1 (4)
C10—N2—C18	120.3 (4)	C12—C11—C10	118.7 (5)
C10—N2—Pd1	107.9 (3)	C12—C11—H11	120.7
C18—N2—Pd1	129.2 (3)	C10—C11—H11	120.7
N1—C1—C2	119.9 (4)	C11—C12—C13	120.8 (5)
N1—C1—C6	120.1 (4)	C11—C12—H12	119.6
C2—C1—C6	119.9 (5)	C13—C12—H12	119.6
C3—C2—C1	119.4 (5)	C12—C13—C14	123.4 (5)
C3—C2—H2	120.3	C12—C13—C18	117.9 (5)
C1—C2—H2	120.3	C14—C13—C18	118.7 (5)
C2—C3—C4	121.6 (5)	C15—C14—C13	120.7 (5)
C2—C3—H3	119.2	C15—C14—H14	119.6
C4—C3—H3	119.2	C13—C14—H14	119.6
C5—C4—C3	118.8 (5)	C14—C15—C16	120.3 (5)
C5—C4—H4	120.6	C14—C15—H15	119.9
C3—C4—H4	120.6	C16—C15—H15	119.9
C4—C5—C6	122.2 (5)	C17—C16—C15	120.9 (5)
C4—C5—H5	118.9	C17—C16—H16	119.6
C6—C5—H5	118.9	C15—C16—H16	119.6
C7—C6—C5	123.6 (5)	C16—C17—C18	120.4 (5)
C7—C6—C1	118.3 (5)	C16—C17—H17	119.8
C5—C6—C1	118.0 (5)	C18—C17—H17	119.8
C8—C7—C6	120.6 (5)	N2—C18—C17	121.0 (4)
C8—C7—H7	119.7	N2—C18—C13	120.1 (4)
C6—C7—H7	119.7	C17—C18—C13	118.9 (4)
			
N2—Pd1—N1—C9	−29.4 (3)	C1—N1—C9—C10	−168.6 (4)
Br1—Pd1—N1—C9	141.3 (3)	Pd1—N1—C9—C10	23.2 (5)
Br2—Pd1—N1—C9	36.4 (7)	C7—C8—C9—N1	−1.0 (7)
N2—Pd1—N1—C1	164.1 (4)	C7—C8—C9—C10	174.6 (5)
Br1—Pd1—N1—C1	−25.1 (4)	C18—N2—C10—C11	−6.7 (7)
Br2—Pd1—N1—C1	−130.0 (5)	Pd1—N2—C10—C11	156.5 (4)
N1—Pd1—N2—C10	30.8 (3)	C18—N2—C10—C9	169.1 (4)
Br1—Pd1—N2—C10	−34.3 (8)	Pd1—N2—C10—C9	−27.7 (4)
Br2—Pd1—N2—C10	−138.2 (3)	N1—C9—C10—N2	3.3 (6)
N1—Pd1—N2—C18	−168.0 (4)	C8—C9—C10—N2	−172.6 (4)
Br1—Pd1—N2—C18	126.9 (5)	N1—C9—C10—C11	179.1 (4)
Br2—Pd1—N2—C18	23.0 (4)	C8—C9—C10—C11	3.1 (7)
C9—N1—C1—C2	168.6 (4)	N2—C10—C11—C12	−0.9 (7)
Pd1—N1—C1—C2	−26.1 (6)	C9—C10—C11—C12	−176.4 (4)
C9—N1—C1—C6	−7.3 (6)	C10—C11—C12—C13	6.4 (7)
Pd1—N1—C1—C6	158.0 (3)	C11—C12—C13—C14	174.8 (5)
N1—C1—C2—C3	−176.4 (4)	C11—C12—C13—C18	−4.5 (7)
C6—C1—C2—C3	−0.5 (7)	C12—C13—C14—C15	−175.5 (5)
C1—C2—C3—C4	−2.1 (8)	C18—C13—C14—C15	3.8 (8)
C2—C3—C4—C5	2.2 (8)	C13—C14—C15—C16	−0.7 (8)
C3—C4—C5—C6	0.3 (9)	C14—C15—C16—C17	−1.6 (8)
C4—C5—C6—C7	174.7 (5)	C15—C16—C17—C18	0.6 (7)
C4—C5—C6—C1	−2.8 (8)	C10—N2—C18—C17	−169.1 (4)
N1—C1—C6—C7	1.1 (7)	Pd1—N2—C18—C17	31.7 (6)
C2—C1—C6—C7	−174.7 (4)	C10—N2—C18—C13	8.5 (6)
N1—C1—C6—C5	178.7 (4)	Pd1—N2—C18—C13	−150.7 (4)
C2—C1—C6—C5	2.9 (7)	C16—C17—C18—N2	−179.9 (4)
C5—C6—C7—C8	−172.2 (5)	C16—C17—C18—C13	2.5 (7)
C1—C6—C7—C8	5.3 (7)	C12—C13—C18—N2	−3.0 (7)
C6—C7—C8—C9	−5.3 (8)	C14—C13—C18—N2	177.7 (4)
C1—N1—C9—C8	7.4 (7)	C12—C13—C18—C17	174.7 (4)
Pd1—N1—C9—C8	−160.8 (4)	C14—C13—C18—C17	−4.7 (7)

### Hydrogen-bond geometry (Å, º)

**Table:**

*D*—H···*A*	*D*—H	H···*A*	*D*···*A*	*D*—H···*A*
C2—H2···Br1	0.95	2.73	3.252 (5)	116
C14—H14···Br1^i^	0.95	2.90	3.754 (5)	150
C17—H17···Br2	0.95	2.85	3.261 (5)	107

Symmetry code: (i) *x*, *y*, *z*−1.

## References

[bb1] Bruker (2000). *SADABS*, *SMART* and *SAINT* Bruker AXS Inc., Madison, Wisconsin, USA.

[bb2] Farrugia, L. J. (1997). *J. Appl. Cryst.* **30**, 565.

[bb3] Muranishi, Y., Wang, Y., Odoko, M. & Okabe, N. (2005). *Acta Cryst.* C**61**, m307–m310. 10.1107/S0108270105013375 15930674

[bb4] Sheldrick, G. M. (2008). *Acta Cryst.* A**64**, 112–122. 10.1107/S0108767307043930 18156677

[bb5] Spek, A. L. (2009). *Acta Cryst.* D**65**, 148–155. 10.1107/S090744490804362X PMC2631630 19171970

